# Neuroprotective Effects of Alpha-Mangostin on MPP^+^-Induced Apoptotic Cell Death in Neuroblastoma SH-SY5Y Cells

**DOI:** 10.1155/2015/919058

**Published:** 2015-08-18

**Authors:** Prachya Janhom, Permphan Dharmasaroja

**Affiliations:** ^1^Toxicology Graduate Program, Faculty of Science, Mahidol University, Bangkok 10400, Thailand; ^2^Department of Anatomy, Faculty of Science, Mahidol University, Bangkok 10400, Thailand

## Abstract

*In vitro* studies have shown that extracts from mangosteen (*Garcinia mangostana* Linn.) act as antioxidants and cytoprotective agents against oxidative damage. The protective effect of alpha-mangostin, the major xanthone found in the pericarp of the mangosteen, in cellular models of Parkinson's disease (PD), has not been investigated. This study aims to investigate whether alpha-mangostin could protect SH-SY5Y neuroblastoma cells from MPP^+^-induced apoptosis. The effects of alpha-mangostin on MPP^+^-induced cell death were evaluated with a cell viability assay, staining for nuclear DNA morphology, flow cytometry for apoptotic cells and reactive oxygen species (ROS) production, quantitative real-time PCR for the expression of p53, Bax, and Bcl-2, and western blot analysis for cleaved caspase-3. Concomitant treatment with alpha-mangostin attenuated the effect of MPP^+^ on cell viability and apoptotic cell death. Alpha-mangostin reduced ROS formation induced by MPP^+^. Bax/Bcl-2 expression ratio and expression of p53 were significantly lower in cells cocultured with alpha-mangostin and MPP^+^. The cotreated cells showed a significant decrease in activated caspase-3 compared with MPP^+^ treatment alone. Our data suggest that cytoprotection of alpha-mangostin against MPP^+^-induced apoptosis may be associated with the reduction of ROS production, modulating the balance of pro- and antiapoptotic genes, and suppression of caspase-3 activation.

## 1. Introduction

Parkinson's disease (PD) is a neurodegenerative disorder characterized by the progressive degeneration of dopaminergic neurons in the substantia nigra pars compacta, resulting in the subsequent loss of function of the basal ganglia circuit. The molecular pathogenesis of PD is believed to be associated with mitochondrial dysfunction, oxidative stress, and activation of the apoptotic cascade [1]. The synthetic compound 1-methyl-4-phenyl-1,2,3,6-tetrahydropyridine (MPTP) induces permanent parkinsonism in humans via its metabolite MPP^+^ (1-methyl-4-phenylpyridinium) [2, 3]. MPP^+^ has been shown to induce a PD-like pathology in animals and cellular models by selective and potent inhibiting of complex 1 of the mitochondrial electron transport chain [4, 5]. MPP^+^-induced neuronal death is mediated by impairment of the mitochondrial membrane potential and opening of the mitochondrial permeability transition pore [6, 7]. Elevation in the reactive oxygen species (ROS) level has also been involved in MPP^+^-induced cytotoxicity [8–10]. Activation of the apoptotic cascade may play a role in MPP^+^-induced cell death by altering mitochondrial membrane permeability and controlling the release of cytochrome c from mitochondria [11, 12]. Caspase-3 activation by released cytochrome c has been shown to involve MPP^+^-induced apoptosis [9, 13, 14]. Once activated, caspase-3 will induce nuclear DNA condensation and fragmentation and, ultimately, apoptosis [15].

A number of antioxidants, such as xanthones, have been demonstrated to have a protective effect on vulnerable neurons under oxidative stress conditions [16–18]. The fruit hull of mangosteen (*Garcinia mangostana* Linn.), a tropical fruit, has been demonstrated to exert an antioxidative effect. The fruit hull contains various xanthone derivatives including alpha-mangostin. Alpha-mangostin was shown to induce a protective effect in cardiac reperfusion damage by attenuation of oxidative stress [19]. Neuroprotective activities of alpha-mangostin against H_2_O_2_-induced oxidative stress have been demonstrated in NG108-15 neuroblastoma cells [20]. This xanthone ameliorated iodoacetate-induced cell death in primary cultures of cerebellar granule neurons by reducing ROS formation [21]. Alpha-mangostin was also shown to attenuate the neurotoxicity induced by beta-amyloid oligomers in SK-N-SH neuroblastoma cells and primary rat cerebral cortical neurons [22, 23]. The antioxidative property of alpha-mangostin is probably mediated by its modulatory effect on the activity of glutathione peroxidase [24]. Although alpha-mangostin has been reported to possess potential neuroprotective properties, there is insufficient information on its protective effects in a PD cellular model. This study aims to investigate whether alpha-mangostin could protect SH-SY5Y neuroblastoma cells from MPP^+^-induced apoptosis and the possible underlying mechanisms.

## 2. Materials and Methods

### 2.1. Cell Culture

The SH-SY5Y human neuroblastoma cells were cultured in a 1 : 1 mixture of Dulbecco's Modified Eagle Medium (DMEM) and Nutrient Mixture Ham's F12 medium and supplemented with 10% heat-inactivated fetal bovine serum (FBS), 1 mM sodium pyruvate, 0.1 mM nonessential amino acid, 1.5 g/L sodium bicarbonate, 100 units/mL penicillin, and 100 *μ*g/mL streptomycin. All media and supplements were purchased from Gibco (Gaithersburg, MD, USA). Cells were maintained at 37°C in a humidified atmosphere of 5% CO_2_. In the experiment, cells were subcultured and plated onto appropriate culture plates. The number of cells to be subcultured was assessed under a phase-contrast microscope based on the exclusion of trypan blue dye. The cultured cells were maintained for 2 days to allow for adhering on the plates. Thereafter, cells were treated with *α*-mangostin (Sigma-Aldrich, St. Louis, MO, USA), MPP^+^ (Sigma-Aldrich, St. Louis, MO, USA), or a combination of *α*-mangostin and MPP^+^ according to the experiment design.

### 2.2. Measurement of Cell Viability

SH-SY5Y cells were seeded onto a 96-well plate at a density of 8 × 10^3^ cells/well in 200 *μ*L of medium and incubated at 37°C under 5% CO_2_ in a humidified incubator for 2 days. After exposure to MPP^+^ (1000 *μ*M), *α*-mangostin (10 *μ*M), or a combination of both for 24 hours, cell viability was measured by MTT (3-(4,5-dimethylthiazol-2-yl)-2,5-diphenyltetrazolium bromide) colorimetric assay (Sigma-Aldrich, St. Louis, MO, USA). This method was based on the reduction of tetra ring of MTT by mitochondrial dehydrogenases with NADH in the active mitochondria, yielding a blue formazan product, which can be measured spectrophotometrically. After incubation, 20 *μ*L of MTT (5 mg/mL) was added to each well and the cells were cultured for another 4 hours, then medium was removed, and 100 *μ*L of DMSO (Sigma-Aldrich, St. Louis, MO, USA) was added to each well to dissolve the formazan. The color reaction was measured at wavelength 570 nm with a reference at 690 nm using The VERSAmax Tunable microplate reader with SoftMax Pro software (Molecular Devices, Sunnyvale, CA, USA).

### 2.3. Staining of Nuclear DNA

SH-SY5Y cells were seeded on coverslips and fixed with 4% paraformaldehyde in 0.01 M PBS (pH 7.4) for 20 minutes at room temperature and then rinsed three times with PBS. After being washed with PBS, the cells were stained with 1 mg/mL Hoechst 33258 (Sigma-Aldrich, St. Louis, MO, USA) in PBS for 20 minutes at room temperature and then washed again. Coverslips were then mounted on slides with DABCO mounting medium. Hoechst 33258 (bisbenzimide) preferentially binds to triplet adenine and thymine base pairs in the minor groove outside the double helix, which allows one to observe the morphological change in the nuclei of apoptotic cells. Nuclear morphology was examined under a laser scanning confocal microscopy (Olympus FV1000, Olympus, Tokyo, Japan) with excitation wavelength 556 nm and emission wavelength 573 nm.

### 2.4. Flow Cytometric Detection of Apoptotic Cells

SH-SY5Y cells were seeded onto 6-well plates at a density of 12 × 10^4^ cells/well in 2 mL of medium and then incubated at 37°C under 5% CO_2_ in a humidified incubator for 24 hours. After exposure to MPP^+^ (1000 *μ*M), *α*-mangostin (10 *μ*M), or the combination of MPP^+^ (1000 *μ*M) and *α*-mangostin (10 *μ*M) for 24 hours, cells were trypsinized and centrifuged at 2,500 rpm, 4°C for 5 minutes. The pellet was washed twice with cold PBS and resuspended at a concentration 1 × 10^6^ cells/mL. Apoptotic cells were detected using an FITC annexin-V/dead cell apoptosis kit (Molecular Probes, Eugene, OR, USA). Cells were incubated with 5 *μ*L of FITC annexin-V and 1 *μ*L of the 100 *μ*g/mL propidium iodide (PI) at room temperature for 15 minutes. The apoptotic cells were analyzed with a FACSCalibur flow cytometer (BD Biosciences, San Jose, CA, USA), measuring the fluorescence emission at 530 nm (FL1) and >575 nm (FL3). Both early apoptotic (annexin-V-positive and PI-negative) and late apoptotic (annexin-V-positive and PI-positive) cells were included in cell death determinations.

### 2.5. Flow Cytometric Detection of Intracellular ROS

The production of ROS was determined by measuring the intensity of fluorescence emitted by cell-permeable fluorescent dyes, dihydrorhodamine 123 (DHR123) and dihydroethidium (DHE), purchased from Invitrogen (Eugene, OR, USA). DHR123 is oxidized to a fluorescent rhodamine-123 by intracellular hydrogen peroxide and peroxynitrite, while DHE is oxidized by superoxide anion to ethidium. SH-SY5Y cells were seeded on 6-well plates at a density of 12 × 10^4^ cells/well and incubated at 37°C under 5% CO_2_ in a humidified incubator for 48 hours. After exposure to MPP^+^ (1000 *μ*M), *α*-mangostin (10 *μ*M) or the combination of MPP^+^ (1000 *μ*M) and *α*-mangostin (10 *μ*M) for 48 hours, cells were washed twice with Hanks buffer salt solution (HBSS) and trypsinized by 0.25% Trypsin/EDTA and centrifuged at 2,000 rpm for 7 minutes. The pellets were incubated with 15 *μ*M DHR123 and 10 *μ*M DHE for 20 minutes at 37°C in the dark. Thereafter, cells were washed with HBSS before cell fixation in 1% paraformaldehyde and the mean fluorescence intensity (MFI) of green (FL1, DHR) or red (FL2, DHE) fluorescence was determined using a FACSCalibur flow cytometer.

### 2.6. Quantitative Real-Time PCR Analysis

SH-SY5Y cells were seeded onto 6-well plates at a density of 12 × 10^4^ cells/well and incubated at 37°C under 5% CO_2_ in a humidified incubator for 24 h. After exposure to MPP^+^ (1000 *μ*M), *α*-mangostin (10 *μ*M) or the combination of MPP^+^ (1000 *μ*M) and *α*-mangostin (10 *μ*M) for 24 hours, cells were trypsinized and centrifuged at 2,500 rpm, 4°C for 5 minutes. Total mRNA was extracted from the pellet using PARIS kit according to the supplier's instructions. The quantity and purity of RNA were determined by optical density measurements at OD A260/A280 ratio with 1.8 or above using Nanodrop 2000 spectrophotometer (Thermo Fisher Scientific Inc., Wilmington, DE, USA). The integrity of RNA was confirmed by running 0.5 *μ*g of RNA samples on 1% agarose gel. Later, the cDNA was synthesized from 1 *μ*g of RNA using Masterscript RT-PCR System (5 PRIME, Gaithersburg, MD, USA), according to the manufacturer's instruction and stored at −20°C until assay. KAPA SYBR FAST qPCR kit (Kapa Biosystems, Woburn, MA, USA) was used for real-time PCR quantification. The 20 *μ*L Real-time PCR reaction mixture contained 20 ng cDNA template, 10 *μ*L of 1x KAPA SYBR FAST qPCR master mix, 200 nM of forward and reverse primers, and PCR-grade water. *β*-Actin was used as a reference gene. The sequences of the primers for the qRT-PCR are as follows: p53: sense, 5′-GGAGGTTGTGAGGCGCTGG-3′; antisense, 5′-CACGCACCTCAAAGCTGTTC-3′; Bax: sense, 5′-CCCGAGAGGTCTTTTTCCGAG-3′; antisense, 5′-CCAGCCCATGATGGTTCTGAT-3′; Bcl-2: sense, 5′-CATGTGTGTGGAGAGCGTCAA-3′; antisense, 5′-GCCGGTTCAGGTACTCAGTCA-3′; *β*-actin: sense, 5′-TGCAGAGGATGATTGCTGAC-3′; antisense, 5′-GAGGACTCCAGCCACAAAGA-3′. The reaction was performed in performed in the Applied Biosystems 7500 Real-time PCR system (Applied Biosystems, Foster City, CA, USA) with the PCR cycling conditions as follows: 3 minutes enzyme activation at 95°C, 40 cycles of 3 seconds initial denaturation at 95°C and annealing/extension at 58°C for 32 sec. Melting curve analysis was performed for verify specificity of each primer after PCR to ensure amplification specificity. The threshold cycle (Ct) number was determined and used in the comparative Ct method. The relative quantity of the target gene was estimated by the 2^−ΔΔCt^ method. All data was analyzed by the ABI 7500 software, version 2.0.

### 2.7. Western Blot Analysis for Cleaved Caspase-3

SH-SY5Y cells were plated into 6-well plates at a density of 12 × 10^4^ cells/well and incubated overnight. After exposure to MPP^+^ (1000 *μ*M), *α*-mangostin (10 *μ*M) or the combination of MPP^+^ (1000 *μ*M) and *α*-mangostin (10 *μ*M) for 24 hours under 5% CO_2_ in a humidified incubator at 37°C, cells were trypsinized by 0.25% Trypsin/EDTA and centrifuged at 4,000 rpm, 4°C for 5 minutes. The pellets were washed with cold PBS, resuspended and incubated on ice for 30 minutes. Then cells were vortexed and centrifuged at 15,000 rpm for 15 minutes at 4°C. The supernatant was collected and total protein concentration was determined using a BCA protein assay. An equal amount of 30 *μ*g proteins from each experiment group were separated by 15% SDS-polyacrylamide gel electrophoresis. The membrane was blocked with a 5% Skim milk for 2 hours, washed with TBST buffer, and incubated with 1 : 1000 dilution of cleaved caspase-3 primary antibodies (Cell Signaling Technology, Denvers, MA, USA) or 1 : 5000 dilution of monoclonal *β*-actin primary antibodies (Sigma-Aldrich, St. Louis, MO, USA) overnight at 4°C. After washing, the membrane was incubated with 1 : 10000 dilution of HRP-conjugated goat polyclonal anti-rabbit IgG (Abcam, Cambridge, UK) as the secondary antibody for cleaved caspase-3 and 1 : 5000 HRP-conjugated goat anti-mouse IgG (Invitrogen, Eugene, OR, USA) as the secondary antibody for *β*-actin for 2 hours at room temperature before being developed using ECL Prime Western Blotting Detection (GE Healthcare, Buckinghamshire, UK). The membrane was reproved with anti *β*-actin antibody to control for equal loading of protein. The signal intensities were determined by densitometry using Image-J software (National Institutes of Health, Bethesda, Maryland, USA).

### 2.8. Statistical Analysis

All experiments were performed in triplicate (*n* = 3). Statistical analyses were performed with one-way ANOVA test followed by a post hoc analysis (Tukey's multiple comparison test) using GraphPad Prism 5 Software for Windows (GraphPad Software, Inc., San Diego, CA, USA). All values were presented as mean ± standard error of the mean (mean ± SEM) for each group. *P* < 0.05 was considered statistical significant.

## 3. Results

### 3.1. Effect of Alpha-Mangostin on MPP^+^-Induced Viability Loss in SH-SY5Y Cells

**Figure 1:**
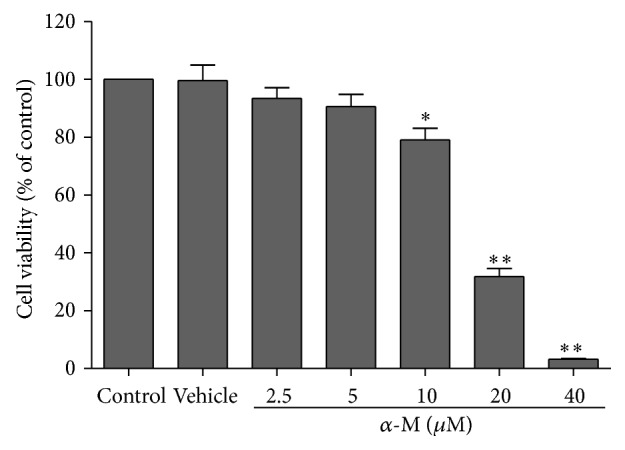
Effect of alpha-mangostin on cell viability of SH-SY5Y cells. After exposure of cells to various concentrations of alpha-mangostin (*α*-M) for 24 hours, cell viability was assessed with MTT assay. Vehicle was DMSO that was used to dissolve MTT. Data are expressed as mean ± SEM (*n* = 3) of percentage to a control.  ^*∗*^ *P* < 0.01;  ^*∗∗*^ *P* < 0.001.

**Figure 2:**
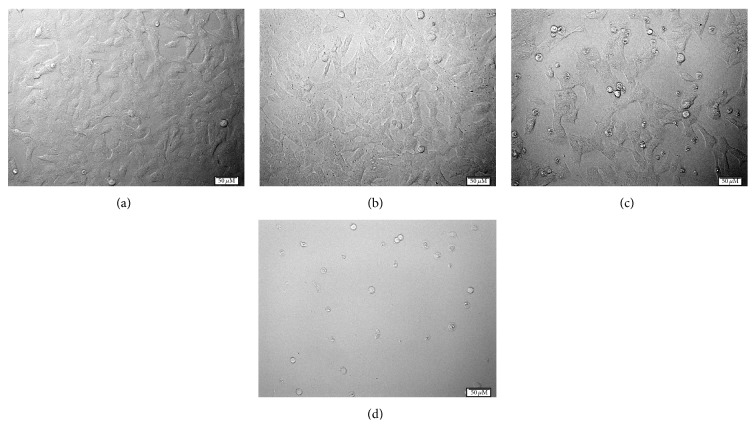
Phase-contrast images of SH-SY5Y cells exposed to various concentrations of alpha-mangostin. After exposure of cells to 0 (a), 10 (b), 20 (c), and 40 *μ*M (d) of alpha-mangostin for 24 hours, cell viability was assessed with phase-contrast microscopy.

**Figure 3:**
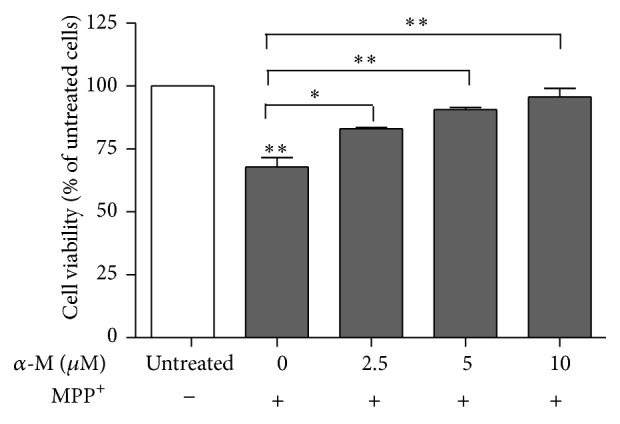
Effect of alpha-mangostin on cell viability of MPP^+^-treated SH-SY5Y cells. Cells were exposed to 0, 2.5, 5, and 10 *μ*M of alpha-mangostin (*α*-M) in the presence of 1000 *μ*M MPP^+^ for 48 hours. Data are expressed as mean ± SEM (*n* = 3) of percentage to untreated cells.  ^*∗*^ *P* < 0.01;  ^*∗∗*^ *P* < 0.001.

To investigate the influence of alpha-mangostin on neuronal cell viability, we treated SH-SY5Y cells with various concentrations of alpha-mangostin for 24 hours and examined cell viability with the MTT assay. Exposure of SH-SY5Y cells to alpha-mangostin induced a reduction in cell viability in a concentration-dependent manner. Cell viability significantly decreased when cells were treated with 10 *μ*m alpha-mangostin for 24 hours, compared with that of control (*P* < 0.01; Figures 1 and 2). Treatment of 20 and 40 *μ*m alpha-mangostin led to loss of cell viability by more than 50% (*P* < 0.001; Figure 1). When SH-SY5Y cells were exposed to 2.5–10 *μ*m alpha-mangostin in the presence of 1000 *μ*m MPP^+^ for 48 hours, the co-treated cells showed a significant increase in cell viability in a concentration-dependent manner, compared to those treated with 1000 *μ*m MPP^+^ alone (Figure 3). Based on these results, 10 *μ*m alpha-mangostin was utilized in later experiments.

### 3.2. Effect of Alpha-Mangostin on MPP^+^-Induced Apoptosis in SH-SY5Y Cells

**Figure 4:**
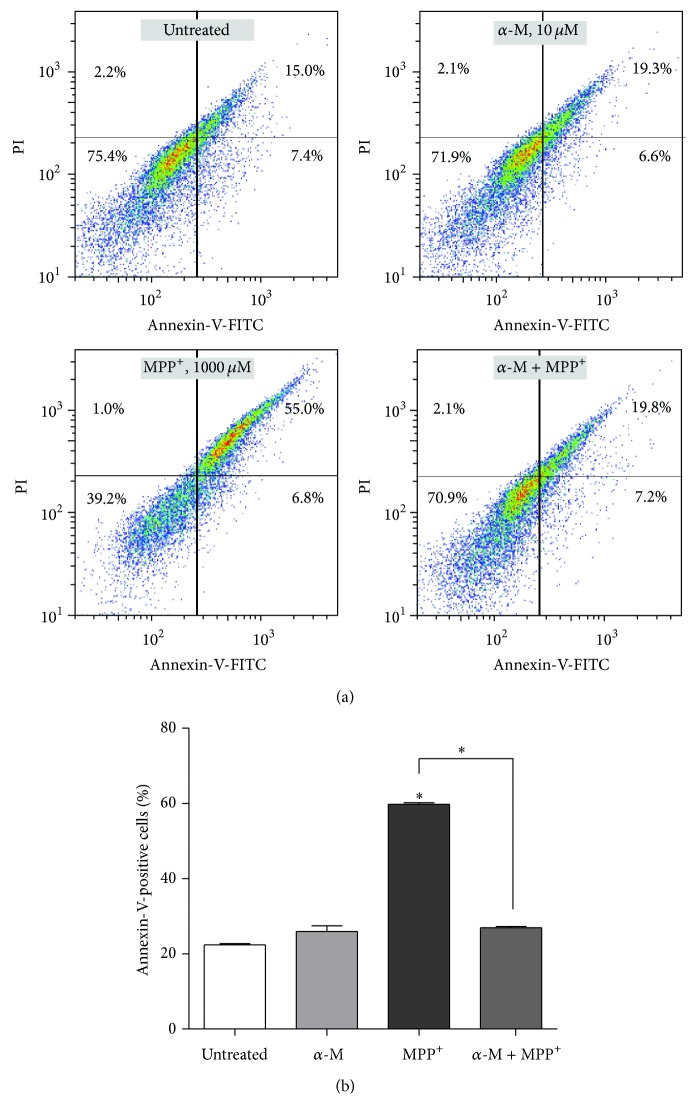
Effect of alpha-mangostin on the MPP^+^-induced apoptosis in SH-SY5Y cells. Cells were exposed to 10 *μ*M of alpha-mangostin (*α*-M), 1000 *μ*M MPP^+^, or a combination of *α*-M and MPP^+^ for 24 hours. Apoptosis was evaluated by staining cells with annexin-V. Flow cytometry profile represents annexin-V-FITC staining in *x*-axis and PI in *y*-axis (a). The number in the right upper quadrant (annexin-V^+^/PI^+^ cells) represents the percentage of late apoptotic/necrotic cells in each condition. Percentage of annexin-V-positive cells was calculated (b). Data are expressed as mean ± SEM (*n* = 3) of percentage to untreated cells.  ^*∗*^ *P* < 0.001.

**Figure 5:**
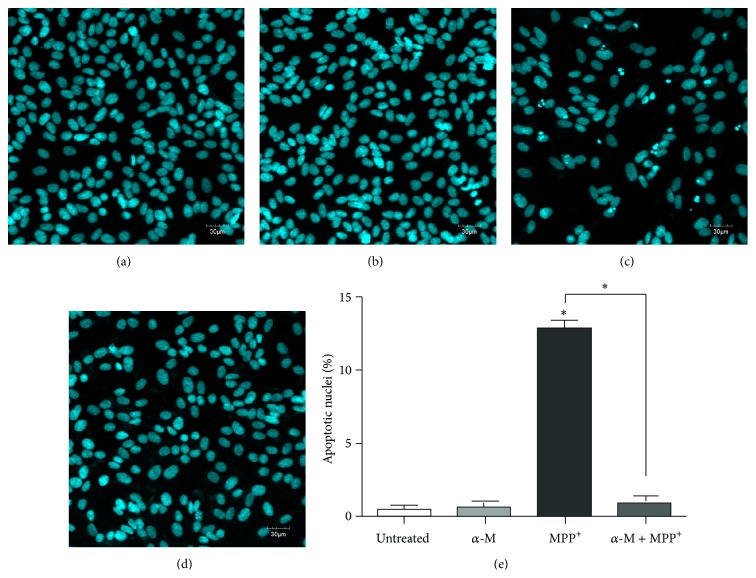
Effect of alpha-mangostin on nuclear morphology in MPP^+^-treated SH-SY5Y cells. Control cells were untreated (a). Treated cells were exposed to 10 *μ*M of alpha-mangostin (b), 1000 *μ*M MPP^+^ (c), or a combination of *α*-M and MPP^+^ (d) for 24 hours. Apoptotic nuclear morphology was visualized by DNA staining with Hoechst 33258. Percentage of cells with apoptotic nuclei was calculated (e). Data are expressed as mean ± SEM (*n* = 3) of percentage to untreated cells.  ^*∗*^ *P* < 0.001.

Apoptotic cells were quantified with PI and annexin-V dual staining using flow cytometry. The annexin-V^−^/PI^−^ population consisted primarily of normal healthy cells, while annexin-V^+^/PI^−^ cells were considered to be in the early stage of apoptosis, and annexin-V^+^/PI^+^ cells were those considered to be in the necrosis/late apoptosis stage (Figure 4(a)). After exposure to 1000 *μ*m MPP^+^ for 24 hours, the percentage of apoptotic cells increased (*P* < 0.001; Figure 4(b)). Cotreatment with 10 *μ*m alpha-mangostin in the presence of 1,000 *μ*m MPP^+^ for 24 hours significantly alleviated the apoptosis when compared to those treated with MPP^+^ only (*P* < 0.001). Nuclear morphology characteristic of apoptosis was further investigated using Hoechst 33258 staining. Condensed or fragmented nuclei were characterized as apoptotic nuclei. Treatment with 10 *μ*m alpha-mangostin decreased the number of apoptotic nuclei (Figure 5). Alpha-mangostin significantly decreased the percentage of apoptotic nuclei when compared to MPP^+^ treatment alone (*P* < 0.001; Figure 5(e)).

### 3.3. Effect of Alpha-Mangostin on MPP^+^-Induced Elevation in the Intracellular ROS Level

**Figure 6:**
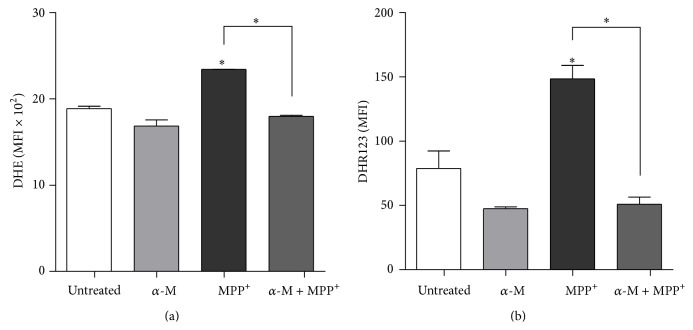
Effect of alpha-mangostin on the MPP^+^-induced ROS production in SH-SY5Y cells. Intracellular ROS production was measured after treatment cells with 10 *μ*M alpha-mangostin (*α*-M), 1000 *μ*M MPP^+^, or the combination of *α*-M and MPP^+^ for 6 hours. Flow cytometric analysis of ROS production is presented as the mean fluorescence intensity (MFI), as assessed by incubation with DHE (a) and DHR123 fluorescence dye (b). Data are expressed as mean ± SEM (*n* = 3), compared to untreated cells.  ^*∗*^ *P* < 0.001.

To evaluate whether ROS play an important role in the attenuation effect of alpha-mangostin on MPP^+^-induced apoptosis in SH-SY5Y cells, cells were exposed to 1000 *μ*m MPP^+^ and 10 *μ*m alpha-mangostin plus 1000 um MPP^+^ for 6 hours, and intracellular ROS production was assessed as DHE and DHR123 fluorescence using flow cytometry. The result shows that cotreatment with 10 *μ*m alpha-mangostin in the presence of 1000 *μ*m MPP^+^ significantly decreased intracellular ROS levels as measured by the mean fluorescence intensity (MFI) of DHE (*P* < 0.001; Figure 6(a)) and DHR123 (*P* < 0.001; Figure 6(b)), compared to MPP^+^ treatment alone.

### 3.4. Effect of Alpha-Mangostin on Bax, Bcl-2, and p53 mRNA Expression in MPP^+^-Treated SH-SH5Y Cells

**Figure 7:**
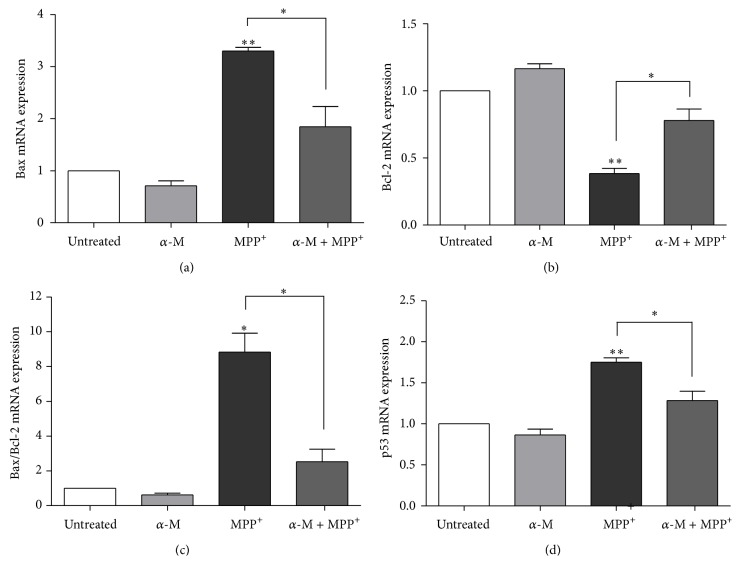
Effect of alpha-mangostin on Bax, Bcl-2, and p53 mRNA expression in MPP^+^-treated SH-SY5Y cells. Cells were treated with 10 *μ*M alpha-mangostin (*α*-M), 1000 *μ*M MPP^+^, or the combination of *α*-M and MPP^+^ for 24 hours. Expression of Bax (a), Bcl-2 (b), Bax/Bcl-2 ratio (c), and p53 mRNA (d) was analyzed with quantitative real-time RT-PCR, relatively compared to their respective untreated controls. The expression levels of the target gene were estimated by the 2^−ΔΔCt^ method after normalizing to the expression level of *β*-actin. Data are expressed as mean ± SEM (*n* = 3).  ^*∗*^ *P* < 0.01;  ^*∗∗*^ *P* < 0.001.

Expression of mRNA of Bax and Bcl-2 was investigated using real-time quantitative RT-PCR analysis. Bax expression significantly increased in 1000 *μ*m MPP^+^-treated cells, compared to that of control cells (Figure 7(a)). Cotreatment of 10 *μ*m alpha-mangostin and 1000 *μ*m MPP^+^ for 24 hours significantly decreased the Bax expression, compared to MPP^+^ treatment alone (*P* < 0.01). Treatment with 1000 *μ*m MPP^+^ significantly decreased Bcl-2 expression, while cotreatment with 10 *μ*m alpha-mangostin significantly increased Bcl-2 expression (*P* < 0.01; Figure 7(b)). As a result, Bax/Bcl-2 expression was significantly increased in SH-SY5Y cells treated with 1000 *μ*m MPP^+^ for 24 hours (*P* < 0.001; Figure 7(c)), whereas Bax/Bcl-2 expression was significantly lowered in cells cocultured in 10 *μ*m alpha-mangostin and 1000 *μ*m MPP^+^ (*P* < 0.001). An upstream regulation of Bax and Bcl-2 was then investigated. The expression of p53 mRNA was significantly increased in 1000 *μ*m MPP^+^-treated SH-SY5Y cells after 24-hour exposure when compared to unexposed cells (*P* < 0.001; Figure 7(d)). Cotreatment of 10 *μ*m alpha-mangostin and 1000 *μ*m MPP^+^ significantly reduced p53 expression when compared to cells cultured in MPP^+^ alone (*P* < 0.01).

### 3.5. Effect of Alpha-Mangostin on Activated Caspase-3 Protein in MPP^+^-Treated SH-SH5Y Cells

**Figure 8:**
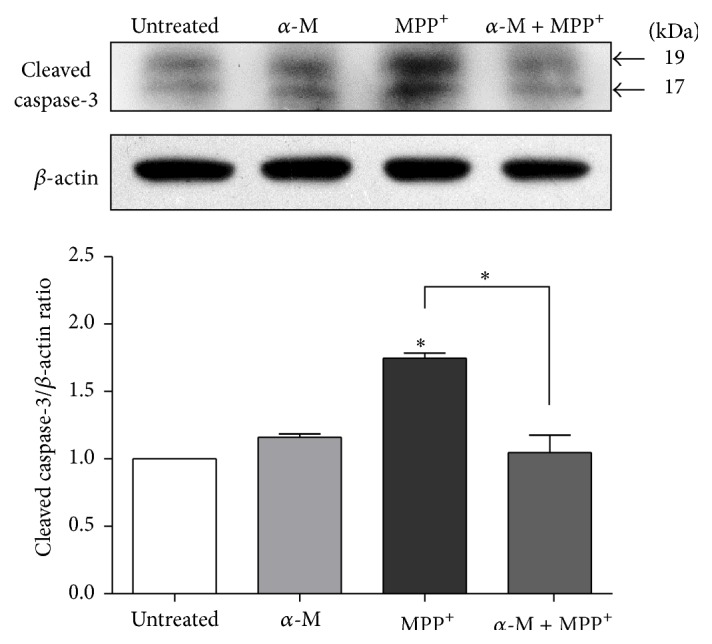
Effect of alpha-mangostin on cleaved caspase-3 protein expression in MPP^+^-treated SH-SY5Y cells. Cells were treated with 10 *μ*M alpha-mangostin (*α*-M), 1000 *μ*M MPP^+^, or the combination of *α*-M and MPP^+^ for 24 hours. Cleaved caspase-3 was detected with Western blotting. The density of bands was analyzed in comparison with that of *β*-actin. Data are expressed as mean ± SEM (*n* = 3).  ^*∗*^ *P* < 0.01.

To investigate the effect of alpha-mangostin on the caspase-3 protein, a critical executioner of apoptosis, a Western blot analysis was conducted. Activation of caspase-3 requires cleavage of the protein at Asp175 into the activated p17 and p19 fragments. After a 24-hour treatment of SH-SY5Y cells with 1000 *μ*m MPP^+^, a significant increase in activated caspase-3 protein was detected compared to the level in control cells (Figure 8). When SH-SY5Y cells were exposed to 10 *μ*m alpha-mangostin in the presence of 1000 *μ*m MPP^+^, the cotreated cells showed a significant decrease in activated caspase-3 compared to the MPP^+^ treatment alone (*P* < 0.001).

## 4. Discussion

The present study has, for the first time, demonstrated that alpha-mangostin rescues apoptosis in dopaminergic SH-SY5Y cells treated with MPP^+^, a cellular model of Parkinson's disease. The results suggest that cytoprotection of alpha-mangostin against MPP^+^-induced apoptosis may be associated with the reduction of ROS production, modulating the balance of pro- and antiapoptotic genes, and suppression of caspase-3 activation.

MPP^+^ is a neurotoxin used to generate animal and cellular models of Parkinson's disease. Several studies have shown that MPP^+^ exerts oxidative stress on cells. MPP^+^ has been shown to increase ROS in neuroblastoma cells [9, 25, 26] and induce superoxide anion (O_2_^−^) production via inhibition of mitochondrial complex I [10, 27]. The presence of antioxidant enzymes such as superoxide dismutase protects against MPP^+^ toxicity in neuronal cell lines [28] and dopaminergic neurons in primary culture [29]. Previous studies showed that alpha-mangostin reduced ROS formation [21, 24, 30]. In the present study, DHE and DHR123 were used to assess ROS production in neuroblastoma cells. DHE is used extensively to monitor superoxide production, perhaps the most specific dye that is retained well by cells [31, 32]. Superoxide radical is the earliest free radical generated as a result of MPP^+^ exposure. Our results suggest that alpha-mangostin attenuated the oxidative effect of MPP^+^ through the reduction of superoxide production. Superoxide anion is converted by intracellular superoxide dismutase to hydrogen peroxide (H_2_O_2_). DHR123 is used for the detection of peroxide and peroxynitrite (ONOO^−^) [32].* In vitro*, alpha-mangostin is a potent scavenger of ONOO^−^ and O_2_^−^ [33]. Our DHR123 oxidation assay suggests that treatment with alpha-mangostin also decreased the production of H_2_O_2_ and ONOO^−^ induced by MPP^+^.

Several studies have supported the role of ROS as intermediates of apoptosis signaling. ROS are elevated in cells undergoing apoptosis [34] and antioxidants have been shown to protect neuronal cells against apoptosis induced by a variety of apoptotic agents [35]. ROS activate caspase-3 and caspase-3-like proteases in various cell types including neuronal cells, leading to nuclear condensation and DNA fragmentation [36, 37]. ROS also induce apoptosis, probably by decreasing expression of Bcl-2, an antiapoptotic molecule [9, 38]. Bcl-2 and Bax may control the mitochondrial permeability transition pore, which can influence the passage of cytochrome c and other apoptosis-inducing factors that trigger the activation of caspase cascade and result in apoptosis [9]. Mangosteen extract has been shown to protect SK-N-SH neuroblastoma cells against an A*β*-induced increase in caspase-3 activity [22]. Here, we showed that alpha-mangostin decreased caspase-3 activation, decreased Bax mRNA expression, and increased Bcl-2 mRNA expression induced by MPP^+^ in dopaminergic SH-SY5Y neuroblastoma cells.

A previous study on cisplatin-induced apoptotic death showed that alpha-mangostin attenuates the increase in p53 expression induced by cisplatin [39]. The connection between p53 and Bcl-2 family members has been established [40]. Proapoptotic Bax, directly induced by p53, can overcome the antiapoptotic effect of Bcl-2, whereas p53 can directly inhibit Bcl-2. In the present study, treatment with 1000 *μ*m MPP^+^ significantly increased the expression of p53. As a result, increased p53 induced Bax expression and inhibited Bcl-2 expression, leading to the apoptosis of SH-SY5Y cells. Our results suggest that alpha-mangostin might reduce the effect of MPP^+^ on Bax and Bcl-2 expression by attenuating p53 expression.

Caspase-3 has been shown to be involved in the apoptotic events occurring in the mitochondrial-dependent pathway, which are associated with nuclear condensation and DNA cleavage [41]. Caspase-3 also induces phosphatidylserine externalization from the internal to external leaflets of the plasma membrane [42]. Phosphatidylserine exposure on the external leaflet of the plasma membrane can be bound with annexin-V and is widely observed during apoptosis [43]. Our DNA staining and PI/annexin-V dual staining supported the role of alpha-mangostin in protection against nuclear changes and phosphatidylserine externalization induced by MPP^+^ treatment in SH-SY5Y neuroblastoma cells.

Alpha-mangostin is an antioxidant among the most abundant bioactive xanthones found in the mangosteen pericarp, which has long been used in traditional medicine to treat diarrhea, dysentery, infected wounds, and chronic ulcers [30]. It has a broad range of bioactivities, such as antioxidant, anti-inflammation, anticancerogenic, and antihistaminergic effects [39, 44–47]. Alpha-mangostin has a cardioprotective effect against myocardial infarct and reperfusion injury in rats; such an effect was related to low levels of lipid peroxidation, a decrease in protein carbonylation and the preservation of high glutathione content [19, 48]. Recently, alpha-mangostin has been shown to inhibit and dissociate the beta-amyloid aggregation, which could contribute to its effect of attenuating beta-amyloid oligomers-induced neurotoxicity in a cellular model of Alzheimer's disease [23].

## 5. Conclusion

This study indicates that alpha-mangostin protects SH-SY5Y cells against MPP^+^-induced apoptosis. The underlying mechanism could be attributed to its antioxidative properties and thus modulating the apoptotic process. The results showed that alpha-mangostin potentially possesses neuroprotective effects in a cellular model of PD. Alpha-mangostin is a small lipid-soluble molecule with the potential to pass the blood-brain barrier [49]. Thus, it may be a promising candidate in the treatment of PD. However,* in vivo* studies for clinical data should be explored.
